# Phasome analysis of pathogenic and commensal *Neisseria* species expands the known repertoire of phase variable genes, and highlights common adaptive strategies

**DOI:** 10.1371/journal.pone.0196675

**Published:** 2018-05-15

**Authors:** Joseph J. Wanford, Luke R. Green, Jack Aidley, Christopher D. Bayliss

**Affiliations:** Department of Genetics and Genome Biology, University of Leicester, Leicestershire, United Kingdom; Emory University School of Medicine, UNITED STATES

## Abstract

Pathogenic Neisseria are responsible for significantly higher levels of morbidity and mortality than their commensal relatives despite having similar genetic contents. Neisseria possess a disparate arsenal of surface determinants that facilitate host colonisation and evasion of the immune response during persistent carriage. Adaptation to rapid changes in these hostile host environments is enabled by phase variation (PV) involving high frequency, stochastic switches in expression of surface determinants. In this study, we analysed 89 complete and 79 partial genomes, from the NCBI and Neisseria PubMLST databases, representative of multiple pathogenic and commensal species of Neisseria using Phasome*It*, a new program that identifies putatively phase-variable genes and homology groups by the presence of simple sequence repeats (SSR). We detected a repertoire of 884 putative PV loci with maxima of 54 and 47 per genome in gonococcal and meningococcal isolates, respectively. Most commensal species encoded a lower number of PV genes (between 5 and 30) except *N*. *lactamica* wherein the potential for PV (36–82 loci) was higher, implying that PV is an adaptive mechanism for persistence in this species. We also characterised the repeat types and numbers in both pathogenic and commensal species. Conservation of SSR-mediated PV was frequently observed in outer membrane proteins or modifiers of outer membrane determinants. Intermittent and weak selection for evolution of SSR-mediated PV was suggested by poor conservation of tracts with novel PV genes often occurring in only one isolate. Finally, we describe core phasomes—the conserved repertoires of phase-variable genes—for each species that identify overlapping but distinctive adaptive strategies for the pathogenic and commensal members of the *Neisseria* genus.

## Introduction

### The genus *Neisseria*

Pathogenic members of the *Neisseria* genus are a major cause of morbidity and mortality worldwide. Within-host development of genetic variation is thought to be key to both the pathogenic and commensal behaviour of this genus. As most host colonisation events are clonal, localised hypermutation resulting in phase variation (PV) could be a major contributor to the genetic and phenotypic variation present within individual hosts.

The *Neisseria* genus consists of two human pathogens (*N*. *meningitidis*; meningococcus, and *N*. *gonorrhoeae*; gonococcus) and several commensals, some of which have caused rare cases of disease [1,2]. Encapsulated meningococci are responsible for severe invasive infections including septicemia and meningitis. The exact mechanisms underlying crossing of the mucosal barrier into the blood, and further transcytosis across the blood-brain barrier are not known. Gonococci are a major cause of genitourinary infections such as urethritis, and can progress to prostatitis and epididymitis in men, and pelvic inflammatory disease in women. The most well-known and frequently-isolated human-specific organisms of this genera are *N*. *meningitidis*, *N*. *gonorrhoeae* and *N*. *lactamica*. The other species, such as *N*. *elongata*, are infrequently observed as commensals of humans and occasionally are isolated from other host organisms, such as *N*. *musculi* from mice. It is likely, however, that the diversity and host range of this genus will expand as exploration of other host species is intensified.

The capacity of the *Neisseria* genus to act as human commensals and pathogens is derived from an arsenal of colonisation, and virulence factors. These include those involved in adhesion to epithelial surfaces, immune resistance and iron acquisition. The ability to evade the immune response by generating antigenic variation within these elements is thought to be a major survival strategy of the genus that facilitates host persistence. PV due to high frequency, reversible mutations in simple sequence repeats (SSR) is one prevalent mechanism of antigenic variation.

### Phase variation and the phasome

PV involves stochastic switching of gene expression from an ‘ON’ to an ‘OFF’ phase by affecting translation or stepped alterations in the level of transcription between arbitrarily-defined low, medium and high phase states. The periodicity of switching is controlled by the underlying mechanism (i.e. mutation, recombination or epigenetic) and usually exceeds 1x10^-5^ mutations per division. There are several specific mechanisms of PV, which have previously been reviewed elsewhere [3–5].

The main mechanism of PV found in *Neisseria* is mediated by mutations in hyper-mutable, SSRs. These SSRs can be found within the open reading frame (ORF) of a given gene or within the promoter region. During genome replication, these sequences are prone to insertion or deletion of repeat units through slip strand mispairing (SSM). The number of repeats in a tract correlates with mutability of the loci, whereby a higher repeat number leads to increased mutability and vice versa [6]. Also, repeat units with longer sequences require fewer repeats to generate high mutation rates [7]. PV in multiple genes can quickly give rise to a wide variety of antigenically unique progeny that are derived from a single ancestral cell and have an almost identical genetic content. The combinatorial phase states derived from multiple PV genes are termed the phasotype [8,9]. The number of phasotypes is the factorial of the number of phase states for each locus and the number of phase variable genes resulting in rapid access to a significant diversity space.

In addition to the significant diversity within populations, there is a large amount of diversity arising from having numerous phase-variable genes within a species and the genus. This diversity across strains and species is known as the phasome. Understanding the consequences of variability generated by phase-variable gene expression in tandem with detailed analysis of reported literature describing the biological function of these genes is essential to understand how Neisseria succeed as human pathogens and commensals.

### Current understanding of PV in Neisseria

The currently sequenced genomes of Neisseria are approximately 2.2 megabases in size, have a G/C content of 50%, and share much of their genetic content [10–12]. The shared genetic content is thought to represent a shared evolutionary lineage and/or colonization parameters. In Neisseria, understanding of PV is heavily biased towards the meningococcus and gonococcus. In this context we have some understanding of the role of PV in evasion of the immune response, iron scavenging, and adhesion to host surfaces [4,13,14]. For the commensal species, the types, number and mutability of the phase-variable genes is poorly understood. Exploration of the phasomes of these species will generate new ideas on the generic roles for specific phasotypes and classes of phase-variable genes in persistent colonisation of the human mucosa and indicate whether interactions between the commensals and pathogenic *Neisseria can* influence the ability of these pathogens to cause disease.

In this study, we have analysed genomes of both commensal and pathogenic members of the genus *Neisseria*, outlining the extent of repeat-mediated PV in this genus, and alluding to the functional consequences that this variation mechanism may have on Neisserial disease and commensalism.

## Methods

### Genome selection and annotation

Complete genomes were extracted from NCBI while partial genomes were extracted from the Neisseria PubMLST database. Partial genomes were chosen for extraction if the assembled sequence data exceeded 2 Mb, indicative of a near full genome size. Strains were selected to give a diverse range of sequence types for each species. Species identify was confirmed by Multi Locus Sequence Typing analysis [15].

Complete genome sequences of *N*. *meningitidis* (n = 68), *N*. *gonorrhoeae* (n = 9), *N*. *lactamica* (N = 1), *N*. *elongata* (n = 1), *N*. *sicca* (n = 1), and *N*. *weaveri* (n = 2) were extracted as GenBank files. Partial genome sequences of *N*. *lactamica* (n = 9), *N*. *elongata* (n = 4), *N*. *sicca* (n = 6), *N polysaccharea* (n = 9), *N*. *mucosa* (n = 5), *N*. *flavescens* (n = 7), *N*. *weaverii* (n = 5), *N*. *cinerea* (n = 5), *N*. *subflava* (n = 5), *N*. *wadsworthii* (n = 1), *N*. *shayganii* (n = 1), *N*. *bacilliformis* (n = 4), *N*. *meningitidis* (n = 10), and *N*. *gonorrhoeae* (n = 10) were extracted as FASTA files and analysed in PROKKA to compare and annotate genes using the genome sequence of *N*. *meningitidis* strain MC58 as a reference. Genome sequences used in this study are shown in S1 Table.

### Analysis of genome sequences using Phasome*It*

**Fig 1:**
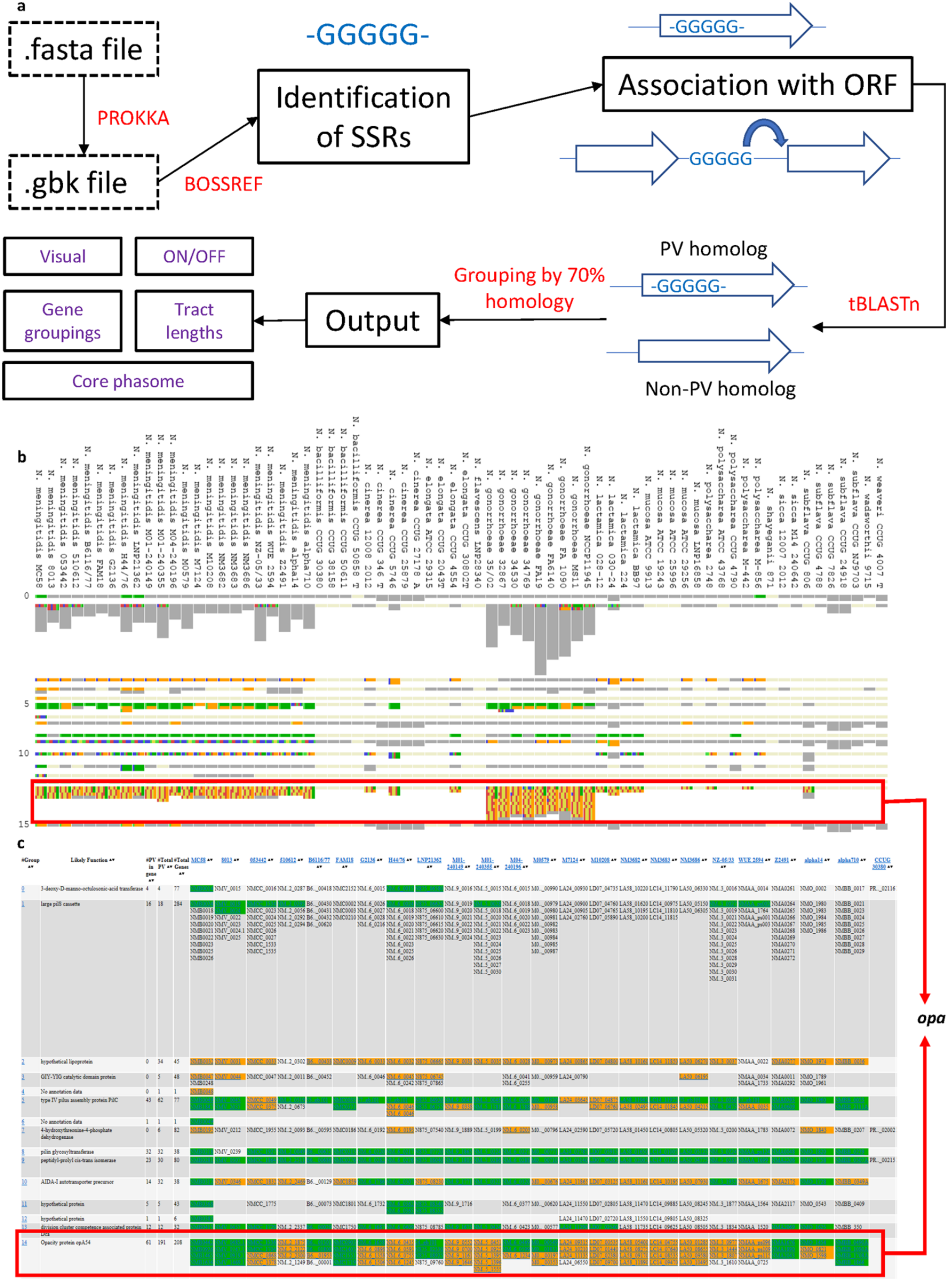
Flowchart and visual output of Phasome*It*. **(A)** Outputs of Phasome*It* can be viewed visually on the index page. Green bars indicate there is an homopolymeric tract within the open reading frame; orange bars indicate there is an SSR close to the gene of interest (for example in a promoter region); grey bars indicate there is a non-PV gene homologous to a PV gene in that same homology grouping; the remaining coloured bars are indicative of SSRs other than homopolymers which can be further derived from the dataset below the visual output. **(B)** Gene groupings corresponding to the visual output are found in a table below. From here, functions, PV status in each strain and tract entries can be obtained for the grouping of interest. The full dataset from which this figure is derived, containing further phasome information not discussed in this manuscript are available (https://figshare.com/s/d31b7b0b6ca4aeeb48df). A red outline shows highlights both the graphical and interactive outputs for the *opa* loci as an example.

The Phasome*It* program has been described by Aidley *et al*. (under review). This program was designed to detect genes subject to PV due to hypermutable SSR. A brief overview is provided herein and a flowchart in Fig 1a. Phasome*It* is written in the Python coding language and can be invoked by Python 3.5.0. Identification of SSRs by Phasome*It* is performed by the Bossref script, which identifies all SSRs in the genome sequences using specific cut-offs for repeat number for each repeat unit type. Phasome*It* utilises positional data from Bossref to associate all SSRs with an ORF (henceforth defined as a PV gene). Phasome*It* classifies PV genes as translational if the SSR is intragenic and transcriptional if the SSR is located within a putative promoter region (i.e. within 200 bp of the 5’ATG). The SSR is designated as intergenic if it is located distant from the 5’ end of a gene or between the 3’ ends of two genes. For translational PV genes, all six reading frames are interrogated by Phasome*It* to identify potential frameshifts (resulting from SSM) and the longest ORF beginning with an ATG codon (designated as the ON state). Homologs of each PV gene are identified using a translated nucleotide search and compared by BLAST to all other genomes in the dataset to identify PV and non-PV homologs. Homologs are clustered into PV homology groups if any PV gene in the group exhibits an amino acid subject coverage of >50%, a query coverage of >40%, and an E value of 10^−6^, to another PV or non-PV gene. Secondary outputs of Phasome*It* include: the core phasome, which comprises all PV homology groups present in 60% or more of genomes of each sub-set (e.g. a species); neighbor-joining and UPMGA phylogenetic trees based on both presents/absence of homology groups or ON/OFF expression states of common PV genes.

For the Neisserial genomes, an initial analysis of each separate genome was performed in Phasome*It* in order to assess the integrity of data arising from partial genome sequences (i.e. were a comparable number of PV genes identified in both complete and partial genome assemblies). All genomes used in this analysis, are shown in the S1 Table.

Repeat numbers indicative of phase variability for a given locus were chosen based on the potential for similarities in mutability of given repeat types. These numbers were: poly-G/C tracts, ≥9; poly-T/A tracts, ≥11; dinucleotide and trinucleotide tracts, ≥8; tetranucleotide and pentanucleotide tracts, ≥5; and tracts consisting of SSRs between unit lengths 5 and 9, ≥3.

### Other bioinformatics analyses

Further interrogation of PV genes was performed using NCBI BLAST. Candidacy of phase variability in loci of interest obtained from the output of Phasome*It* was further investigated by sequence alignment using ClustalOmega of homologous loci deposited in BIGSdb. For *N*. *meningitidis*, sequences were compared against meningococcal genomes deposited by ‘Bayliss’ (S2 Table), and for other members of the genus, sequences were aligned against the complete, respective species database.

## Results and discussion

Localised hypermutation has evolved independently in multiple genera of bacteria. The phasome of each species and clonal lineage is likely to have evolved due to the combined effects of clonal expansion of genetically-linked genes, shuffling by horizontal gene transfer, and lineage-specific selection of particular traits. Studying the phasomes of the pathogenic and commensal Neisseria provides an opportunity to study how each of these forces have shaped evolution of localised hypermutation in this genus. A large repertoire of phase-variable genes of Neisseria have previously been explored separately but have not included analysis of the commensal species [7,16,17]. A more comprehensive analysis of the Neisseria phasome has been made possible by the generation of large sets of genome data [18] from multiple isolates of the pathogenic species and a diverse range of commensal species. Using a new program, Phasome*It*, we have analysed the conservation and distribution of phase-variable genes in published genomes of a selection of closed- and partial-genomes of Neisserial species (https://figshare.com/s/d31b7b0b6ca4aeeb48df).

Phasome*It* identifies phase-variable genes by the presence of a hypermutable SSR tract within the reading frame or promoter region of a gene (i.e. translational and transcriptional PV genes, respectively). Phasome*It* also enables analysis of the conservation, distribution and functionality of the hypermutable mechanism (i.e. SSRs) and SSR-mediated PV genes in the context of known phylogenies, distributions and phenotypes of these strains and species.

**Fig 2:**
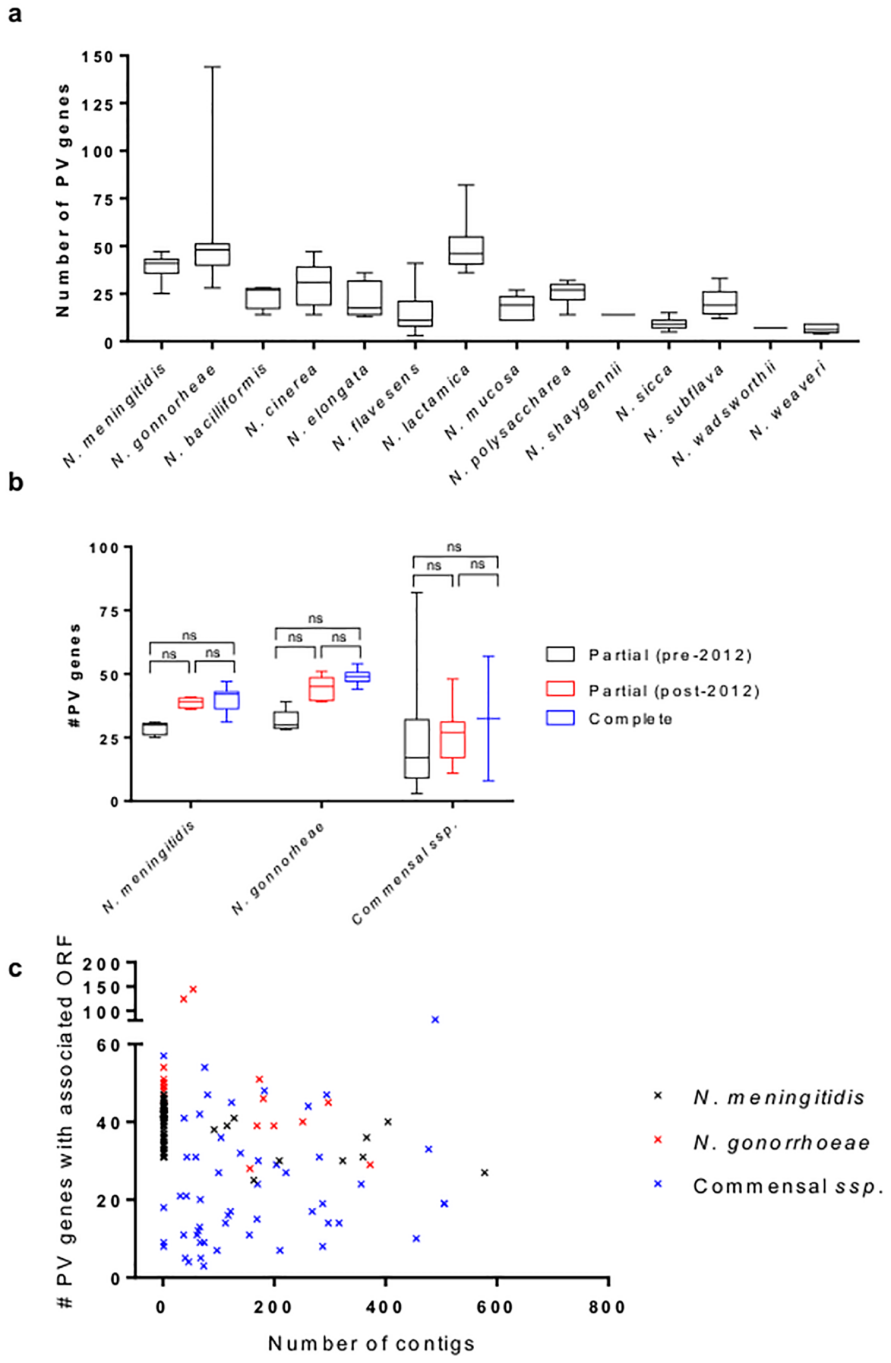
Range of phase variable genes identified in each species. Data shown the median, range, upper and lower quartile number of PV genes, as indicated by presence of a repeat tract. These data exclude gene groupings which contain dinucleotide repeat tracts, due to the insufficient evidence of phase variation associated with dinucleotide repeats in the literature, and the loci discussed herein. Statistical analysis were performed with a Kruskal-Wallis test with Dunn’s multiple comparisons. NS; not significant, ***; p-value of <0.0005.

Analysis of SSR-mediated PV is hampered by the inaccuracies of the next generation sequencing technologies and in particular by sequence read lengths of the Illumina methodology [19]. These potential inaccuracies are particularly relevant to the partial genome sequences. To assess the impact of changes in the Illumina sequencing methodology, we measured the numbers of PV genes in partial genome sequences produced by the Illumina platform from pre- and post-2012, correlating with predominantly shorter and longer read lengths respectively. These values were compared to closed genome sequence data where numbers of PV genes are predicted to be accurate (Fig 2b). For both pathogenic species and a pooled sample of the commensal species, a trend was observed with detection of higher numbers of PV genes in partial post-2012 and closed genome sequences. The ability to detect a greater number of PV genes was associated with reduced contig number in the genome assembly (Fig 2c), due to fewer contig breaks as a result of the relative merit of longer reads in spanning repetitive regions. However, these differences were not significant while post-2012 partial genome sequences and closed genomes exhibited very similar numbers of PV genes. Thus Phasome*It* is a valuable tool for identifying PV genes in both older and more recent sequencing datasets.

### *Neisseria* genomes contain previously unstudied SSRs of diverse composition and repeat number

Cutoffs (see Material and methods) to define a phase-variable repeat tract were selected based on previously reported associations between repeat numbers and phase-variable switches in gene expression for Neisseria where possible, and for other bacterial taxa where SSRs had not been studied in *Neisseria* [8,20]. Where data was not available for a given tract, the cutoff defining a PV repeat was assumed to be the same as those tracts with SSRs of identical unit length and repeat number. Previously, the meningococcal *siaD* gene, has been observed to switch gene expression due to mutations in a repeat tract of G7 [21]. However, *Neisseria* genomes have a high GC content and Phasome*It* identified 3,111 G7 tracts in total, and a mean of 40 G7 tracts per meningococcal genome alone. Moreover, the *siaD* switching rate is <10^−5^ indicating that the mutation rates of these tracts are not high enough to mediate efficient PV [22]. These observations suggest that the *siaD* tract arose by chance due to genome context rather than selection for mutability.

Phasome*It* detected 5,604 SSRs in the 118 Neisseria genomes. As observed previously, a wide range of repeat unit lengths are present ranging from mononucleotides up to nonomeric repeats (Table 1). The predominant unit types identified in this analysis are polyG, polyT and 5’CTTCT repeats accounting for 41%, 8**%**, and 4% respectively of the SSRs identified (https://figshare.com/s/d31b7b0b6ca4aeeb48df). Furthermore, a number of novel repeat units, not previously reported to mediate PV were identified (e.g. 5’ CATTTCT; Table 1), providing an imperative to study the mutability of these loci *in vitro*.

**Table 1:** Variety of repeat types found in pathogenic and commensal *Neisseria ssp*.

Mean number of SSRs per genome (range of repeat numbers)*									
SSR †	*N*.*meningitidis*	*N*.*gonorrhoeae*	*N*.*lactamica*	*N*.*elongata*	*N*.*bacciliformis*	*N*.*cinerea*	*N*.*mucosa*	*N*.*polysaccharea*	*N*.*subflava*
**G**	15.4 (9–26)	18.2 (9–19)	19.4 (9–39)	5 (9–14)	5.8 (9–40)	8.6 (9–33)	6.8 (9–36)	10.2 (9–34)	5.2 (9–36)
**T**	3.6 (11–30)	1.7 (11–16)	4.8 (11–23)	1.8 (11–16)	1.5 (11–14)	4.8 (11–17)	2.2 (11–17)	3.4 (11–41)	2 (11–17)
**GC**	4.3 (6)	4.1 (6)	9.6 (6–7)	2.8 (6)	3.3 (6)	4.3 (6)	2.8 (6–7)	4.6 (6–7)	7.4 (6–7)
**CTTG**	1.4 (5–34)	0.9 (6–14)	1.6 (17–23)	0.2 (23)	0	0.8 (11–15)	0	0.4 (12–23)	0.2 (9)
**CTTCT**	3.2 (5–24)	8.1 (5–21)	1.8 (8)	0	0	0	0.2 (8)	0.6 (8)	0.2 (5)
**CAACCG**	1 (3)	1 (3)	1.2 (3)	0.3 (3)	0	0.6 (3)	0.2 (3)	0.8 (3)	0
**GCGCGT**	0.8 (3)	1 (3)	1.2 (3)	0.3 (3)	0	1.8 (3)	0.2 (3)	0.8 (3)	1.8 (3)
**TAGGCT**	0.7(3)	0.3(3)	0.6(3)	0.3(3)	0	1(3)	0	0.4(3)	0
**CATTTCT**	0.6 (3–22)	1 (10–26)	0	0	0	0.4 (6–14)	0	0	0
**GCCAAAGTT**	0.7 (5–25)	0	0	0	0	0	0	0	0

* The minimum repeat number required for detection of an SSR of a specific repeat unit length or type are as follows: G, 9; T 11; dinucleotides and trinucleotides, 6; tetranucleotides and pentanucleotides, 5; and repeats of between 6 and 9, 3.

^**†**^ Data are not comprehensive but comprise all SSRs that occur at >1% of all the repeats tracts identified in the 118 genomes.

A total of 2,278 poly-G tracts were found in the 118 genomes. The average number of poly-G tracts per genome was significantly different (Table 1) between the meningococcus (15.4) and the gonococcus (18.2; p = >0.05, Kruskal Wallace test) whereas *N*. *lactamica* species averaged 19.4 poly-G tracts which was significantly greater than the meningococcus (p = >0.05; Kruskal Wallace test), but not the gonnococcus. Contrastingly, *N*. *elongata* and all of the other commensal species encode a far lower number of poly-G tracts (1–7), and indeed a lower number of all types of hypermutable sequences (8–35), suggesting a lower level of selection for mutability and a reduced need to generate diversity by PV in these species. This reduced number of tracts in the commensal species may also be indicative of earlier divergence from the pathogenic meningococcus as alluded to by a previous study [23]. Conversely, the closer relationship between repeat tracts in *N*. *lactamica* and the pathogenic species may represent a combination of more recent divergence and on-going lateral gene transfer [24].

An expected difference between the meningococcus and gonococcus was in the number of 5’-CTTCT-3’ repeats (governing expression of the *opa* genes), at means of 3.2 and 8.1 respectively. The actual numbers of expected Opa genes is 4 per meningococcal genome and 11/12 for gonococcal genomes [25] suggesting that repeat tracts are not present in every copy of *opa* in the genome assemblies, or that some opa copies are missing from the assembled sequences due to deletion of from a particular locus or inaccurate assembly of near identical *opa* alleles. Indeed, lower numbers of *opa* alleles were identified in the partial genome sequences of N. *meningitidis*, indicative of assembly problems, and may have perturbed identification of PV *opa* alleles in the many partial genomes of commensal species. *N*. *lactamica* is known to encode up to 2 alleles of *opa* [23,26], and indeed all of the *N*. *lactamica* sequences analysed herein contained 2 PV *opa* alleles, with a single isolate (30770_M45_1) contained an additional non-PV *opa*. This suggests that *opa* genes are not subject to PV in this species but could alternatively indicate that these genes are absent from the partial genome sequences. Consistent with the known role of Opa proteins in pathogenesis [25,27], *opa* genes were not detected in many of the commensal species. However, the presence in some commensal isolates may indicate ongoing gene transfer with pathogenic bacteria and involvement of Opa in colonisation of the niche.

Dinucleotide tracts have previously been shown to facilitate PV in a variety of Gram-negative organisms [28,29], but not in *Neisseria*. Sequence alignments were performed for each dinucleotide tract identified in our study using homologous loci in other isolates (data not shown) but no changes in repeat number were detected confirming absence of putative PV (Table 1).

**Fig 3:**
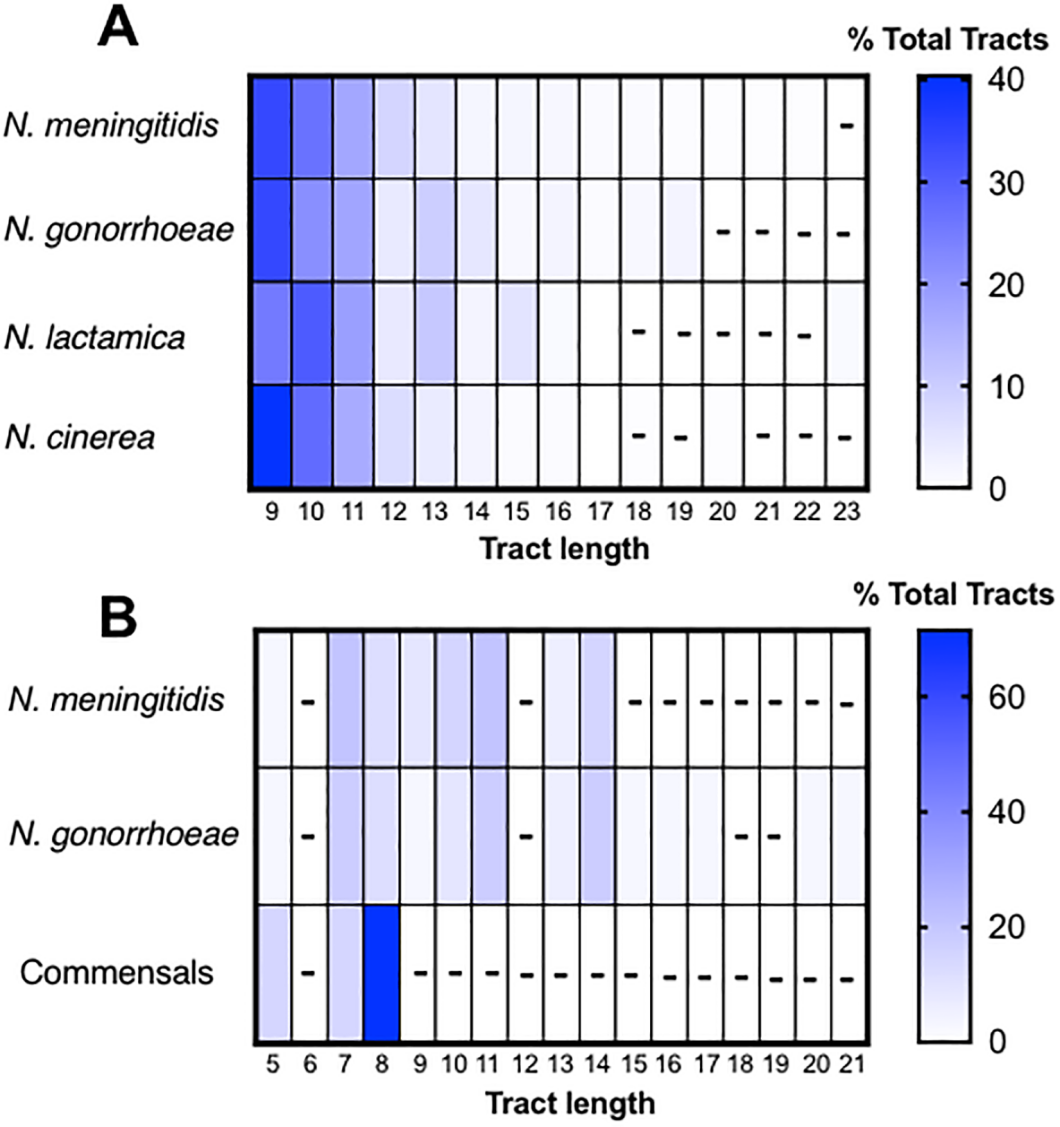
Tract length distribution in different *Neisseria* species. Data are represented by heat maps. Colour intensity represents the percentage that a given tract length comprises of the total number of identified tracts of that type for each species. ‘-’ is indicative of no identified repeats of the given length. Information on the numbers of strains for each species, and numbers of tract lengths analysed can be found in Table 1.

A key feature of PV is the frequency of switching, which is in part determined by the length of the repeat tract. Fig 3 highlights the distribution of repeat numbers of poly-G tracts (Fig 3a) and 5’-CTTCT-‘3 tracts (Fig 3b) in *Neisseria* species. In *N*. *meningitidis*, poly-G tracts of up to length G14 have previously been assayed and increases in mononucleotide tract length correlate with higher PV frequencies [30]. In our analysis, the gonococcus was shown to have a wider distribution of tract lengths (both have a modal tract length of 9), suggesting a minor difference in mutability. As mutation rate is a function of repeat number, these longer poly-G tracts are likely to have mutation rates that are higher than 9.3 x 10^−9^ as detected by Richardson et al. (2002) [30] for a G14 tract in a non-mutator wild-type strain. *N*. *lactamica* showed poly-G tract lengths comparable with the pathogenic species, but a modal repeat length of 10G (Table 1; Fig 3a). These data highlight the potential importance of heightened mutability in facilitating commensalism of *Neisseria* species. The gonococcus was shown to have a greater range of CTTCT tract lengths than the meningococcus (Fig 3b). Exclusion of low repeat numbers in the analysis may have introduced a bias in these data, however short CTTCT tracts were observed in both species. These data may be indicative of a requirement for rapid switches in Opa phenotype for the gonococcus facilitating rapid changes in tropism during infection and frequent immune escape. This hypothesis is further supported by both the absence (or reduced copy number) of *opa* genes in many commensal species (for which we predict the requirement for switching to be low), and in cases where *opa* is present, shorter tract lengths are found (maxima of 8 repeats; Fig 3b) as compared with the pathogenic species.

Finally, whilst published data is available to confirm phase variability of larger length repeat units, such as the heptameric repeats found in *Haemophilus influenzae* high molecular weight adhesins [31], little attention has been paid to the role of repeat numbers of larger length SSRs (greater than pentameric repeats) on PV rates in *Neisseria*. This highlights a need for understanding the mutability associated with the loci with longer length SSRs reported in this study.

### Modular phase variable gene groupings are prevalent in *Neisseria*

Phasome*It* identifies phase variable genes through the presence of a repeat tract, and then assembles these genes into ‘homology groups’ if they display subject coverage of >50%, a query coverage of >40%, and an E value of 10^−6^. This approach facilitates comparison between genome sequences.

Our analysis of 14 *Neisseria* species revealed a total of 884 distinct homology groups arising from 5,604 SSRs indicative of a putative role for phase variability across the genera. This contrasts with previous studies [16] that identified only 115 discreet PV genes. Many of the genes within these groups are only assigned putative functions or have no available annotation in NCBI.

Fig 2a depicts the median number and maximum range of phase-variable genes observed per genome for each species. In the gonococcus, there was a mean of 54 PV genes per genome while the meningococcus contained a statistically significant mean of 39 PV genes (p = <0.05; one way ANOVA with Tukey’s Multiple Comparisons; S3 Table). This difference, may be partially explained by the elevated number of PV *opa* alleles in the gonococcus. Contrastingly the majority of the commensals encoded statistically fewer PV genes when compared to the meningococcus (S3 Table) with the exception of *N*. *lactamica* which had a median number of 48 PV genes, a value comparable with the pathogenic species. Other commensals had significantly lower numbers of PV genes than both the pathogenic *Neisseria* and *N*. *lactamica*, ranging between a minimum of 3 in *N*. *flavensens* and a maximum of 47 in *N*. *cinerea*. Surprisingly, the maximum number of PV genes was found in *N*. *lactamica* 030–24 with a total of 90. Similarities in the PV capabilities of *N*. *lactamica* and the pathogenic *Neisseria* suggest overlapping requirements for this mode of adaptation that may arise from comparable mucosal niches and frequent exposure to immune effectors. Contrastingly, the other commensals may be located in more inert or protected niches such as dental biofilms where adaptability is less frequently required or beneficial.

Five major groups of PV loci in *Neisseria* can be discerned; i) multiple copy OMPS; ii) restriction modification systems; iii) pilin glycosylation/modulation; iv) LPS biosynthesis and; v) single copy OMPs. The majority (53%; 2970 out of 5604) of phase variable genes identified in this analysis mapped to these modules (Table 2). The PV modules contain both phase variable genes and non-PV homologs where the repetitive tract is absent, has been inactivated by mutation or is below the cut-off for a PV SSR. The five major PV modules contain comparable numbers of PV and non-PV homologs suggestive of frequent and on-going selection for PV. Conversely, higher numbers of non-PV homologs compared to PV genes were detected in the hypothetical loci and genes not currently associated with these modules. These ‘sporadic’ PV loci imply a reduced selective advantage for variation or a strain-specific advantage of phase variability for these loci. These sporadic phase variable gene groupings primarily consist of genes involved in metabolism, gene expression and protein degradation, which may form part of an unrecognized functional role of PV or indicate that these SSR have arisen through transient, selection-independent evolution.

**Table 2:** Distribution of phase variable genes between phase variable modules.

PV module	Total PV †	PV in gene ‡	PV intergenic §	Non-PV homologs ||	Ratio PV: non PV #
**Multi-copy OMPs**	389	360	29	121	3.5:1
**RM systems**	248	191	57	119	2:1
**Pilin modulation/glycosylation**	354	320	34	156	2.2:1
**LOS biosynthesis**	147	142	5	79	1.9:1
**Single-copy OMPs**	1498	507	972	2666	1:1.8
**Other**	2335	1245	1109	42402	1:18
**Hypothetical proteins**	633	363	270	15403	1:24
Total	5604	3128	2476	60946	N/A

Due to the high copy number, and sporadic nature of PV in these genes, *pilS* loci were excluded from the pilin analysis. **†** Total PV; the total number of phase variable genes—whether in the ORF or intergenic—mapping to each module. **‡** PV in gene; number of PV genes with repeat tracts in the ORF mapping to each module. **§** PV intergenic; number of PV genes with repeat tracts in intergenic regions. **||** Non-PV homologs; number of genes with homology to genes mapping to each module which do not contain a repeat tract. **#** Ratio; the ratio of genic SSRs + intergenic SSRS: non-PV homologs.

#### PV restriction modification modules

Phase-variable RM systems have been associated with inhibiting phage infection, controlling gene expression and reducing genetic exchange through natural transformation. In *Neisseria*, Type III RM systems alter expression of multiple genes, termed the ‘phasevarion’, and in some strains cause phase-variable expression of virulence determining phenotypes [32,33]. Pathogenic *Neisseria* encode three phase variable RM systems—*modA*, *modB* and *modD*—and each of these genes have several allelic variants with high levels of variation in the target recognition domain. There is also a phase-variable S sub-unit of a Type I RM system that changes the recognition sequence but has no known biological function [34]. RM systems are associated with clonal lineage suggesting a role in restricting DNA transfer and implying that, as in *H*. *pylori* [35], PV of RM systems could vary susceptibility to DNA transfer thereby facilitating adaptation to selective pressures through generation of genetic variation.

Our analysis identified 367 genes encoding RM systems, with 291 of these being subject to translational-PV (Table 2). 103 of these loci were *modA*, with 99 containing a phase variable SSR within the reading frame. A further 127 were *modD* with 70 having translational-SSRs. The remaining RM system genes were *modB*. All 78 meningococcal genomes had *modA* whereas only 65 and 14 had *modD* and *modB* respectively, comparable with previous observations [36]. While the pathogenic species and *N*. *lactamica* isolates encoded between one and three PV RM systems, the other commensal *Neisseria* usually encoded only 1 (51%) or none (49%). This suggests a differential requirement for phase-variable RM systems reflecting reduced benefits arising from impacts on epigenetic switching, natural transformation or phage exposure.

#### Pilin modulation

The type IV pilus of the pathogenic *Neisseria* influences colonization by facilitating adhesion and modulation of epithelial surfaces [37,38]. The *Neisseria* pilus is subject to extensive antigenic variation through recombination events between a functionally expressed *pilE* gene (encoding the major pilus structural protein, PilE) and a series of silent *pilS* loci [39] (Koomey. 1994)(Cahoon and Seifert,m 2011). Silencing of PilE expression can occur following recombination with an inactive *pilS* gene. In addition, the *pilC* genes encode proteins that control retraction of the pilus resulting in modulation of adhesion phenotypes. The pathogenic Neisseria encode two *pilC* genes that are subject to PV due to polyC tracts within the reading frame [40].

Our analysis reveals that 23 of 607 *pilS* alleles contain SSRs (hexameric, CCACCG; nonomeric; CCACCGGCA; homopolymeric, C) and could be subject to in frame and ON/OFF PV respectively. Introduction of SSR-containing *pilS* alleles into the expression locus through homologous recombination may alter not only the protein sequence of *pilE* but also lead to further antigenic variation of the pilus. Interestingly, repeat tracts were only identified in the *pilS* genes of the meningococcus and gonococcus, suggesting that dual mechanisms for generating pilin diversity are only advantageous in these species. Similarly, 145 *pilC* gene sequences, of a total 205, were subject to PV. The *pilC* genes were identified in several of the commensal species, but phase-variable alleles were only found in the pathogens. Seven of the commensal genomes encoded only a single *pilC* gene each. These data indicate that ON/OFF switching of PilC proteins may have only evolved in the pathogenic species of *Neisseria*, possibly as a modulator of host cell adherence. Intergenic recombination may be the primary mechanism for generating diversity in pilin monomers, but ON/OFF switching mediated through introduction of repeat tracts in *pilS* and the *pilC* genes may also have roles in pilus assembly and surface exposure.

#### Pilin glycosylation

An additional mechanism for varying the structure of the *Neisseria* pilus is through PV of genes involved in post-translational glycosylation of pilin [41]. Although the exact role for these different glycosylation states in *Neisseria* biology is unknown, it is thought that switching between some of these states may facilitate immune evasion, while inferences from other species suggest they may play a role in pathogenesis [42,43].

Our analysis identified 356 loci encoding alleles or homologs of the known phase variable pilin glycosyltransferases (105 *pglA*; 129 *pglE*; 122 *pglI*), 209 of which are subject to PV (92 *pglA*; 116 *pglE*; 82 *pglI*). Phase variable pilin glycosyltransferases were found primarily in the pathogenic species and *N*. *lactamica*. Some isolates of *N*. *polysaccharea* and *N*. *mucosa* encoded *pglI* and *pglE* (none of which were subject to PV), but no *pgl* genes (phase variable or otherwise) were detected in the other commensals.

#### Multi-copy outer membrane proteins

By far the most abundant multi-copy outer membrane protein in *Neisseria* which are prone to PV are the opacity-associated proteins (Opa). Opa proteins are thought to play a pivotal role in colonization of the human nasopharynx, tissue tropism and the invasion of host cells/tissues that subsequently leads to invasive meningococcal and gonococcal disease [14,23,44–46]. As a result of a ubiquitous presence and surface exposure, Opa proteins are subject to extensive antigenic variation through PV and recombination involving both intra- and intergenomic events [47]. PV between different Opa proteins facilitates the progression of infection by altering the tissue tropism of the gonococcus [48].

Our data reveal a total of 510 *opa*-family alleles, with 389 subject to PV. In the pathogens, each *opa* locus was subject to PV, with an exception of 6 non-PV variants in the gonococcus and 6 in the meningococcus. These loci contained between 1 and 4 repeats and so fall below the PV cut-off for this unit size. Interestingly, despite the meningococcus being known to encode a maximum of 4 *opa* alleles, we identified two meningococci (DE8669, and MO1-240355) which encoded 5 *opa* loci. *N*. *lactamica* encoded a maximum of 3 PV *opa* alleles (only one of these alleles was not PV) while *N*. *cinerea*, *N*. *elongata* contained a two PV *opa* alleles in 2 and 1 of the isolates respectively, and *N*. *polysaccharea* contained a single PV *opa* loci in 5 of the isolates. No *opa* genes were detected in the other commensal species. These genes may have arisen from horizontal gene transfer with the meningococcus and were observed to have 100% query coverage identity and between 50–60% sequence (BLASTn) identity with *opa* alleles found in meningococci.

#### Lipooligosaccharide biosynthesis

Lipooligosaccharide (LOS) is a key component of the Gram-negative outer membrane. The LOS of *Neisseria*, is highly immunogenic [49] and in this context, PV of genes involved in biosynthesis of surface-exposed epitopes have been shown to mediate escape of killing by specific antibodies [13,50]. *Neisseria* are known to encode multiple PV LOS biosynthesis genes (e.g. *lgtA*, *lgtC*, *lgtD* and *lgtG)* whose switching gives rise to structural distinct immunotypes with differing pathogenic capabilities.

Our analysis identified 266 *lgt* genes, with 147 of these being subject to PV. These genes largely assembled into 2 homology groups due to high levels of sequence conservation between genes. Consistent with the literature, the majority of cognate tracts were poly-G, in the range of 9 to 15 repeats. Similar to the pilin glycosyltransferases, the majority of PV LOS modifying enzymes were present in the pathogens, with sporadic gene groupings mapping to the commensal species.

#### Single-copy outer membrane proteins

Single-copy outer membrane proteins (scOMPs) are ubiquitous throughout bacteria, and exhibit a wide variety of functions, many of which are essential for both persistence in the host and progression to systemic disease. *Neisseria* genomes encode several proteins involved in iron acquisition, such as the two haemoglobin receptors HpuAB and HmbR [51], and the siderophore receptor, FetA [52]. Many iron acquisition systems of *Neisseria* are prone to PV [53], allowing for stochastic switching between a state of iron acquisition and immune evasion [4]. PV of many other scOMPs, such as the outer membrane transporter PorA, contribute to evasion of specific immune responses. Lastly, many scOMPs are involved in epithelial adhesion, such as the surface adhesin NadA whose transcriptional PV gives rise to at least three distinct levels of protein expression [54].

Our data reveal a total of 1,498 phase variable single copy OMPS with 22 homology groups consisting of iron acquisition systems and 30 homology groups for putative adhesins. 10 homology groups encoding efflux systems were identified as being sporadically subject to PV, as were an additional 23 efflux associated proteins. Full details of outer membrane proteins identified in this analysis can be found in (https://figshare.com/s/d31b7b0b6ca4aeeb48df).

### Evidence for a core, species-specific phasome consisting primarily of accessory genes

Consistent with the analysis of Campylobacters by Aidley *et al*. (Under review), the largest fraction of the phase variable genes map to the accessory genome of *Neisseria*. There is however a small number (65 homology groups) of sporadic PV modules with central metabolic functions, such as the tRNA alanyl-synthetase grouping and a putative peptidyl-prolyl cis-trans isomerase (groupings 28 and 9 respectively; https://figshare.com/s/d31b7b0b6ca4aeeb48df). Genes in this category tend to have short, coding SSRs (multiples of 3 nucleotides), and are invariant between strains of a species suggesting strong selection for maintenance of tract length, possibly due to functionality of the amino acids encoded by the tract.

The output of Phasome*It* provides evidence that phase variability is conserved in 90% or more of isolates for several gene groupings (data not shown). These conserved gene groups form a core, species-specific phasome. The core phasomes for *N*. *meningitidis*, *N*. *lactamica* and *N*. *gonorrheae* are comprised of 14, 11, and 14 gene groupings respectively (Table 3). Only 7 of these conserved PV gene groupings however are shared between any two species (for example *N*. *meningitis* and *N*. *lactamica* share 7 common core PV genes, as do *N*. *gonorrheae* and *N*. *lactamica*). For the other commensals where multiple genomes were analysed (note that a core phasome cannot be derived with only a single genome), we were unable to detect a core of phase variable genes in >90% of the genomes or the core phasome consisted of a single gene with an invariant SSR (see https://figshare.com/s/d31b7b0b6ca4aeeb48df).

**Table 3:** Core phasome analysis of a subset of *Neisseria* species.

Gene * ‡	Species § ||			Function	Evidence for PV †	Tract (Range)	Location of SSR
	*N*. *meningitidis*	*N*. *gonorrhoeae*	*N*. *lactamica*				
*modA* (NMB1375)	**++**	**++**	**++**	Restriction modification	Known [36]	GCCA (6–37)	Genic
*modD* (NMB1261)	**+**	**++**	**++**	Restriction modification	Known [36]	CCAATG (7–31)	Genic
NMB2030	**++**	**+**	**++**	Restriction modification	None	GCCGC (3)	Genic
*pglA* (NMB0218)	**++**	**+**	**++**	Pilin glycosylation	Known [42]	G (9–21)	Genic
*pglE* (NMC0568)	**++**	**++**	**+**	Pilin glycosylation	Known [42]	CAAACAC (5–34)	Genic
*pglI* (NMB1836)	**++**	**+**	**+**	Pilin glycosylation	Known [42]	G (9–15)	Genic
*pilC* (NMC0371; *pilC1*)	**+ +**(2)	**++** (2)	**+**	Pilus retraction	Known [40]	G (9–20)	Genic
*Opa*	**++** (4)	**++** (12)	**++** (2)	Adhesion/invasion	Known [14]	CTTCT (7–21)	Genic
*lgtA* (NMB1929)	**+**	**++ (2)**	**+**	LOS biosynthesis	Known [56]	G (9–20)	Genic
*lgtG* (NMC2011)	**+**	**++**	**++**	LOS biosynthesis	Known [56]	C (10–16)	Genic
*fetA* (NMB1988)	**+**	**++**	**-**	Iron acquisition	Known [52]	C (10–14)	Intergenic
NMB0032	**++**	**+**	**++**	Putative lipoprotein	Alignment	A (11–13)	Intergenic
*nalP* (NMB1969)	**++**	**+**	**-**	Autotransporter	Known [57]	C (9–13)	Genic
NMB0468	**++**	**++**	**+**	Arginine decarboxylase	None	TGTTTG (3)	Genic
NMB1460	**++**	**+**	**+**	ssDNA binding protein	None	GCCGC (3)	Genic
NMB1864	**++**	**++**	**++**	Glutamate-1-semialdehyde-2,1-aminomutase	None	CGGTTG (3)	Genic
NMB1352	**++**	**+**	**+**	YSIRK family signal peptide	None	AAGAA (3)	Genic
NMB1595	**++**	**++**	**++**	Alanyl-tRNA synthetase	None	CGCGCC (3)	Genic
*macB* (NGO1439)	**+**	**++**	**+**	Macrolide efflux	Alignment	CAGGG (3–4)	Intergenic
*NMB1605*	**+**	**++**	**++**	Topoisomerase IV subunit A	None	GGCGC (3)	Genic
*tamB* (NMB2135)	**+**	**++**	**+**	Periplasmic protein	None	CCGCC (3)	Genic
NLA_12310	**+**	**+**	**++**	Adhesin	Alignment	C (9–10)	Intergenic

* Genes which were found to be present in greater than 90% of genomes analysed were considered the ‘core phasome’. Phase variable genes present in a smaller percentage of analysed genomes can be found in (https://figshare.com/s/d31b7b0b6ca4aeeb48df). **†** ‘Known’ indicates genes with previous evidence for PV; ‘Alignment’ indicates genes for which there is alignment based evidence in this study; ‘None’ indicates that no switching in repeat length were identified in this analysis. **‡** Where NMB numbers are given, these are in reference to homologs in the meningococcal type strain MC58, where NGO numbers are given, these are in reference to the gonococcal reference strain FA 1090. where NLA numbers are given, these are in reference to the *N*. *lactamica* reference strain 020–06. **§** ++; PV copy present in >90% of genomes, +; PV copy present in <90% of genomes -; gene absent from the genome assembly. Bracketed numbers indicate the maximum copy number of that gene observed in a respective species. **||** In this case, scoring was based on whether a single one of these loci was PV, further information on the SSRs in each of these genes can be found in (https://figshare.com/s/d31b7b0b6ca4aeeb48df).

The core phasomes of *N*. *meningitidis*, *N*. *lactamica* and *N*. *gonorrheae* contained a phase variable copy of the *modA* gene and at least one phase variable *opa* allele. In particular PV of *modA* is ubiquitous in the meningococcus. One possibility is that ModA mediated PV of the cognate regulon is critical for part of the life cycle of this species. There is however variation between strains in the putative TRDs of this gene, which is likely to produce differences in the regulons and hence strain-to-strain differences in the genes subject to stochastic variation in expression. Alternatively, PV of ModA may alter the frequency of natural transformation facilitating adaptation to selective pressures by increasing horizontal gene transfer. The conservation of Opa may reflect a requirement for frequent switching during persistent carriage in order to maintain adhesion to CEACAMs, the ligands for these proteins, in the face of strong selection for loss of expression due to immune selection. An intriguing possibility is that switching of *modA* increases antigenic variation of *opa*, the other conserved PV gene, as another mechanism for overcoming immune selection. The differences in the numbers of phase variable Opa between species could reflect differing lengths of host persistence and exposure to host immune responses. With this view, the gonococcus may exhibit the longest host persistence and strongest immune response with *N*. *lactamica* occupying the opposite extreme.

Only the meningococcal and gonococcal core phasomes contained phase variable copies of *pilC*. These genes are involved in antagonising retraction of the pilus and as a result are probably critical for initial association with the host epithelium. The rationale for conservation of PV is unclear but may reside in maintenance of function in the face of frequent immune selection against expression.

*N*. *lactamica* has a high number of phase variable genes comparable to the meningococcal repertoire. However, the core phasome of *N*. *lactamica* was only one gene larger than meningococcal core phasome. Interestingly, the major disparity between these phasomes, is the absence of *pilC* (present in 6/7 genomes, but PV in none) from the core phasome of *N*. *lactamica*. It is therefore likely that the pilus is subject to greater immune selection during meningococcal host persistence than for *N*. *lactamica*. Conversely, similarities between the meningococcal and *N*. *lactamica* core phasomes may have arisen as a result of occupying the same nasopharyngeal niche and through co-colonisation of this niche leading to consequent horizontal gene transfer that is both ancestral and on-going [55], indeed it has been suggested that divergence of *N*. *lactamica* from the meningococcus may be evolutionarily recent [24].

These data have shown that a number of previously unstudied genes form part of the core phasome. These genes exhibit sequence based evidence of phase variability and include a putative lipoprotein (NMB0032) in the meningococcus and *N*. *lactamica*, a macrolide efflux pump (*macB*) in the gonococcus, and a putative adhesin in *N*. *lactamica*. Presence in the core phasome indicates niche-specific selective pressures for maintaining phase variability of these loci, and as such, future studies should investigate the functional roles of both expression states of these proteins during commensalism and pathogenesis.

Core phasomes were absent from the all of commensal Neisserial species except *N*. *lactamica*. This suggests that PV is unlikely to be a major determinant of the adaptive potential of most the commensal Neisseria. Conversely, the identification of partially-overlapping core phasomes in *N*. *lactamica* and the two pathogenic *Neisseria* species underlines the critical contribution of PV to mucosal colonisation and persistence of these three species. A remarkably high number of overlapping genes is indicative of the strong similarities in the selective pressures encountered by these species. Conversely the non-overlapping PV genes define key niche or behavioural-specific features of these species reflecting differences in genital versus nasopharyngeal tissues, age of the hosts (which may intersect with differing immunological memories), modes of transmission and varying lengths of host persistence. There were only two cases (the absence of *fetA* and *nalP* from *N*. *lactamica*) where a gene was present in the core phasome of one species but was never present or phase variable in another of these species. This finding reaffirms that strong selective pressures are required to maintain variability of the core phasome loci.

### Characterisation of understudied mechanisms of PV in *Neisseria* using Phasome*It*

Mechanisms of genetic diversity in *Neisseria* are not fully understood. Previous studies in yeast [58] and some bacteria, including a small number of pathogenic *Neisseria* genomes [59,60], have indicated that slippage occurs in repeat tracts consisting of multiples of three nucleotides. Our sequence alignments of homologous loci revealed changes in length of these tracts between strains of a species that is evidence for PV. The functional consequences and fitness benefits associated with this variation has not been elucidated in *Neisseria*, and represents a gap in our understanding of the genetics of this genus. A recent study by Siena *et al*. produced evidence of PV in 115 genes of *N*. *meninigitidis* by deep sequencing, and detection of variability in an SSR [61]. All of the genes classified as highly likely to undergo PV, due to high levels of SSR variation, were detected by our study. Conversely, genes classified as weakly phase variable (i.e. due to low variation in the SSR) were not detected by our study, in large part due to these genes having short SSRs below our limits for identification as PV loci. Both studies detected several PV genes with functions in central metabolism, an underexplored area of PV. In the future, experimental evidence, such as provided by Siena *et al* (2016) [61], is required to validate PV in the genes identified herein.

A gene encoding an ATP-dependent Clp protease was shown to be phase variable in *Neisseria* with changes in tract length at homologous loci observed through comparative genome analysis (Table 4). Clp proteases are known to be phase variable in *Campylobacter* ssp. and involved in stress tolerance and virulence of this organism [62]. By analogy, PV of this gene in *Neisseria* may modulate survival of stresses including those imposed by neutrophils and macrophages with important consequences for virulence. Interestingly, the cognate repeat tract of the Clp protease was a hexameric 5’-TGAAGA-3’ repeat. Analysis of the Clp amino acid sequence from *N*. *meningitidis* MC58 reveals that this tract lies within 50 amino acids of the putative ATP binding site of ClpA, implying a similar mechanism as the change of phenotype observed in a previous study by Zhou *et al*. (2012) [59], in which expansion of an SSR diminished ATPase activity of the MutL, leading to a mutator phenotype [63].

**Table 4:** Exemplar gene groupings associated with in frame and read through phase variation.

Gene grouping	PV	Non PV homologues	Associated tracts	Function
Peptidyl—prolyl cis-trans isomerase*	41	104	GCCAAAGCT (**4–25**) AAACTTGCC (**4–16**)	Protein folding [64]
ATP-dependent Clp protease*	14	69	TGAAGA (**3–4**)	Protein degradation [65]
*kdtA* †	4	75	G (**9–10**)	LOS decoration [66]

* Exemplar genes identified with repeat tracts consisting of multiples of 3 nucleotides indicative of ‘in-frame’ phase variation

^**†**^ Exemplar genes identified subject to 3’, ‘read through’ PV.

Another feature is the presence of nonomeric 5’-GCCAAAGCT-3’ repeat tracts in a highly conserved peptidyl-prolyl cis-trans isomerase (homologous to NMB0281; MC58) in all meningococcal isolates. This tract is located outside of any known functional domain, however, variation in this tract could alter the secondary structure of these proteins. Proteins with prolyl isomerase activity are involved in interconversion of cis-trans isomers of peptide bonds in proline. Previously, studies have indicated that proteins with this activity play a role in persistence of the gonococcus in host macrophages, although the exact functional role of this activity is unclear [67].

Finally, Snyder *et al*. (2016) [17], through sequence analysis and NGS of gonococcal strains following passage, has confirmed the presence of 3’ PV in *N*. *gonorrhoeae* predicted to alter the C-terminal sequence of the encoded protein product or lead to unique protein fusions. As an example output of Phasome*It*, we identified an SSR tract within the 3’ end of NMB0014 encoding the KdtA protein that is involved in addition of a KDO residue to lipid A in the early stages of LOS biosynthesis. A G10 repeat tract gives rise to a full-length transcript for *kdtA*. Deletion of one G extends the reading frame by 175 amino acids, but a further deletion to a G8 tract, allows transcription across both NMB0014 and NMB0013, giving rise to a unique protein fusion which may modify the activity of KdtA or lead to addition of Kdo to an alternative structure. To support this, the meningococcus has previously been shown to be functional without the *kdtA* and its conferred modification [66]. Further interrogation of homologous loci in all deposited meningococcal genomes in BIGSdb (as of August 2016), reveal that these repeat tracts appear to be only present (above the threshold to be defined PV) in serogroup B isolates, suggesting serotype specific selection for PV. A phase variable copy of *kdtA* was only found in one commensal genome, *N*. *polysaccharea* M-856, which encoded *kdtA* with a premature stop codon, and was not fused to the downstream gene. The broad breadth of characterisation of PV mechanisms achieved by Phasome*It* provides an imperative for a more in depth investigation of the PV in this genus, for both pathogenic and commensal species (https://figshare.com/s/d31b7b0b6ca4aeeb48df).

## Conclusion

We have demonstrated that pathogenic *Neisseria* encode a greater number of PV genes than the majority of commensal species. One exception is *N*. *lactamica*, which encodes a slightly higher number of PV genes than the pathogenic species, possibly indicative of more diverse selection pressures and tissue tropisms. We have also shown that *N*. *meningitidis*, *N*. *gonorrhoeae* and *N*. *lactamica* possess overlapping and species-specific core phasomes, which may facilitate both effective colonization of their respective niches, and contribute to the pathogenic behaviour of meningococci and gonococci. Finally, we have described additional outputs off Phasome*It*, including examples of ‘in-frame’ and ‘read through’ mechanisms of PV, which will facilitate further analysis of genetic variation in this genus in the future.

## Supporting information

S1 Table Genome sequences analysed in this study. Complete and partial genome sequences were extracted from the NCBI, and pubMLST databases.
(DOCX)

S2 Table Genomes deposited by Bayliss as of 22.02.17. Genomes are available within the Neisseria PubMLST database.
(DOCX)

S3 Table Statistical comparison of differences in number of PV genes by species. Statistical significance were determined by an ordinary one way ANOVA, with Tukey’s multiple comparisons.
(DOCX)
