# Supplementary material for: Phasome analysis of pathogenic and commensal Neisseria species expands the known repertoire of phase variable genes, and highlights common adaptive strategies
Source: PLoS One. 2018 May 15;13(5):e0196675. doi: 10.1371/journal.pone.0196675 (PMC5953494; doi:10.1371/journal.pone.0196675)
Supplement: S1 Table — Complete and partial genome sequences were extracted from the NCBI, and pubMLST databases. (DOCX) [file pone.0196675.s001.docx]

| **Species** | **Strain** | **#Contigs** |
| --- | --- | --- |
| N. meningitidis | MC58 | 1 |
| N. bacilliformis | CCUG 30380 | 1046 |
| N. bacilliformis | CCUG 38158 | 100 |
| N. bacilliformis | CCUG 50611 | 100 |
| N. bacilliformis | CCUG 50858 T | 112 |
| N. cinerea | 12008 2012 | 59 |
| N. cinerea | CCUG 346 T | 356 |
| N. cinerea | CCUG 5746 | 294 |
| N. cinerea | CCUG 25879 | 281 |
| N. cinerea | CCUG 27178 A | 316 |
| N. elongata | ATCC 29315 | 1 |
| N. elongata | CCUG 2043T | 66 |
| N. elongata | CCUG 4554 | 104 |
| N. elongata | CCUG 30802T | 268 |
| N. flavescens | CD-NF1 | 38 |
| N. flavescens | CD-NF2 | 73 |
| N. flavescens | CD-NF3 | 37 |
| N. flavescens | CNF | 67 |
| N. flavescens | LNP28340 | 287 |
| N. flavescens | NRL30031/H210 | 117 |
| N. flavescens | SK114 | 31 |
| N. gonorrhoeae | 35/02 | 1 |
| N. gonorrhoeae | 15698_DGI2 | 37 |
| N. gonorrhoeae | 15935_PID24 | 54 |
| N. gonorrhoeae | 21072_SK_93_1035 | 156 |
| N. gonorrhoeae | 26827_12028_2010 | 169 |
| N. gonorrhoeae | 27090_S339 | 372 |
| N. gonorrhoeae | 32867 | 1 |
| N. gonorrhoeae | 34530 | 1 |
| N. gonorrhoeae | 34769 | 1 |
| N. gonorrhoeae | 48704_SRR3360690 | 180 |
| N. gonorrhoeae | 49195_SRR3361334 | 199 |
| N. gonorrhoeae | 49218_SRR3361357 | 173 |
| N. gonorrhoeae | 52753_Ng01_34 | 251 |
| N. gonorrhoeae | 56773_NG4 | 297 |
| N. gonorrhoeae | FA19 | 1 |
| N. gonorrhoeae | FA6140 | 1 |
| N. gonorrhoeae | FA 1090 | 1 |
| N. gonorrhoeae | MS11 | 1 |
| N. gonorrhoeae | NCCP11945 | 1 |
| N. lactamica | 020-06 | 1 |
| N. lactamica | 028-12 | 123 |
| N. lactamica | 030-24 | 489 |
| N. lactamica | 224 | 104 |
| N. lactamica | 29274_BM68a | 66 |
| N. lactamica | 30770_M45_1 | 80 |
| N. lactamica | 37261_07AZI_T_009 | 75 |
| N. lactamica | 42495_M16_240028 | 182 |
| N. lactamica | 46053_M31_5 | 261 |
| N. lactamica | BB97 | 104 |
| N. meningitidis | 2_120M | 359 |
| N. meningitidis | 420_NG_F26 | 323 |
| N. meningitidis | 446_1000 | 578 |
| N. meningitidis | 644_L93_4286 | 209 |
| N. meningitidis | 898_OX9930715 | 163 |
| N. meningitidis | 8013 | 1 |
| N. meningitidis | 53442 | 1 |
| N. meningitidis | 55157_2171724 | 366 |
| N. meningitidis | 55512_12029_15 | 92 |
| N. meningitidis | 56753_M20933 | 115 |
| N. meningitidis | 57483_24183 | 128 |
| N. meningitidis | 57923_99M | 404 |
| N. meningitidis | 510612 | 1 |
| N. meningitidis | B6116/77 | 1 |
| N. meningitidis | DE8555 | 1 |
| N. meningitidis | DE8669 | 1 |
| N. meningitidis | DE10444 | 1 |
| N. meningitidis | FAM18 | 1 |
| N. meningitidis | G2136 | 1 |
| N. meningitidis | H44/76 | 1 |
| N. meningitidis | L91543 | 1 |
| N. meningitidis | LNP21362 | 1 |
| N. meningitidis | M01-240149 | 1 |
| N. meningitidis | M01-240355 | 1 |
| N. meningitidis | M04-240196 | 1 |
| N. meningitidis | M0579 | 1 |
| N. meningitidis | M7124 | 1 |
| N. meningitidis | M07149 | 1 |
| N. meningitidis | M07161 | 1 |
| N. meningitidis | M07162 | 1 |
| N. meningitidis | M08000 | 1 |
| N. meningitidis | M08001 | 1 |
| N. meningitidis | M09261 | 1 |
| N. meningitidis | M09293 | 1 |
| N. meningitidis | M10208 | 1 |
| N. meningitidis | M12752 | 1 |
| N. meningitidis | M22160 | 1 |
| N. meningitidis | M22189 | 1 |
| N. meningitidis | M22191 | 1 |
| N. meningitidis | M22718 | 1 |
| N. meningitidis | M22722 | 1 |
| N. meningitidis | M22740 | 1 |
| N. meningitidis | M22745 | 1 |
| N. meningitidis | M22748 | 1 |
| N. meningitidis | M22759 | 1 |
| N. meningitidis | M22769 | 1 |
| N. meningitidis | M22772 | 1 |
| N. meningitidis | M22783 | 1 |
| N. meningitidis | M22801 | 1 |
| N. meningitidis | M22804 | 1 |
| N. meningitidis | M22809 | 1 |
| N. meningitidis | M22811 | 1 |
| N. meningitidis | M22819 | 1 |
| N. meningitidis | M22822 | 1 |
| N. meningitidis | M22828 | 1 |
| N. meningitidis | M23413 | 1 |
| N. meningitidis | M24705 | 1 |
| N. meningitidis | M24730 | 1 |
| N. meningitidis | M25070 | 1 |
| N. meningitidis | M25087 | 1 |
| N. meningitidis | M25419 | 1 |
| N. meningitidis | M25438 | 1 |
| N. meningitidis | M25456 | 1 |
| N. meningitidis | M25459 | 1 |
| N. meningitidis | M25462 | 1 |
| N. meningitidis | M25472 | 1 |
| N. meningitidis | M25474 | 1 |
| N. meningitidis | M25476 | 1 |
| N. meningitidis | M27559 | 1 |
| N. meningitidis | NM3682 | 1 |
| N. meningitidis | NM3683 | 1 |
| N. meningitidis | NM3686 | 1 |
| N. meningitidis | NZ-05/33 | 1 |
| N. meningitidis | WUE2121 | 1 |
| N. meningitidis | WUE 2594 | 1 |
| N. meningitidis | Z2491 | 1 |
| N. meningitidis | alpha14 | 1 |
| N. meningitidis | alpha710 | 1 |
| N. mucosa | ATCC 9913 | 67 |
| N. mucosa | ATCC 19243 | 287 |
| N. mucosa | ATCC 25996 | 61 |
| N. mucosa | ATCC 29256 | 221 |
| N. mucosa | LNP16858 | 155 |
| N. polysaccharea | 2748 | 43 |
| N. polysaccharea | 41528_M15_240955 | 204 |
| N. polysaccharea | 41652_2748 | 221 |
| N. polysaccharea | 42468_M16_240183 | 170 |
| N. polysaccharea | 42472_M15_240827 | 171 |
| N. polysaccharea | ATCC 43768 | 297 |
| N. polysaccharea | CCUG 4790 | 139 |
| N. polysaccharea | M-442 | 221 |
| N. polysaccharea | M-856 | 42 |
| N. shayeganii | 871 | 112 |
| N. sicca | 4320 | 169 |
| N. sicca | 12007 2012 | 74 |
| N. sicca | ATCC 29256 (2) | 61 |
| N. sicca | DS1 | 455 |
| N. sicca | FDAARGOS_260 | 1 |
| N. sicca | M14 240642 | 68 |
| N. sicca | VK64 | 210 |
| N. subflava | CCUG 806 | 477 |
| N. subflava | CCUG 4788 | 63 |
| N. subflava | CCUG 7826 | 505 |
| N. subflava | CCUG 24918 | 505 |
| N. subflava | CCUG NJ9703 | 121 |
| N. wadsworthii | 9715 | 97 |
| N. weaveri | ATCC 51223 | 40 |
| N. weaveri | CCUG 4007 T | 821 |
| N. weaveri | LMG 5135 | 46 |
| N. weaveri | NCTC13585 | 1 |
| N. weaveri | NCTC13585 (2) | 1 |

**Supplementary 1**. Genome sequences analysed in this study. Strain names, and the number of contigs comprising the assembly are shown.
