# Supplementary material for: Phasome analysis of pathogenic and commensal Neisseria species expands the known repertoire of phase variable genes, and highlights common adaptive strategies
Source: PLoS One. 2018 May 15;13(5):e0196675. doi: 10.1371/journal.pone.0196675 (PMC5953494; doi:10.1371/journal.pone.0196675)
Supplement: S2 Table — Genomes are available within the Neisseria PubMLST database. (DOCX) [file pone.0196675.s002.docx]

| **id** | **isolate** | **country** | **continent** | **source** | **epidemiology** | **species** | **serogroup** | **ENA_accession** | **date_entered** |
| --- | --- | --- | --- | --- | --- | --- | --- | --- | --- |
| 28247 | N241.1 | UK | Europe | throat swab | carrier | Neisseria meningitidis | Y | ERR144489 | 2014-03-06 |
| 28248 | N51.1 | UK | Europe | throat swab | carrier | Neisseria meningitidis | Y | ERR144490 | 2014-03-06 |
| 28249 | N52.1 | UK | Europe | throat swab | carrier | Neisseria meningitidis | Y | ERR144491 | 2014-03-06 |
| 28250 | N54.1 | UK | Europe | throat swab | carrier | Neisseria meningitidis | Y | ERR144492 | 2014-03-06 |
| 28251 | N58.1 | UK | Europe | throat swab | carrier | Neisseria meningitidis | Y | ERR144493 | 2014-03-06 |
| 28252 | N59.1 | UK | Europe | throat swab | carrier | Neisseria meningitidis | Y | ERR144494 | 2014-03-06 |
| 28253 | N88.1 | UK | Europe | throat swab | carrier | Neisseria meningitidis | Y | ERR144495 | 2014-03-06 |
| 28254 | N138.1 | UK | Europe | throat swab | carrier | Neisseria meningitidis | Y | ERR144496 | 2014-03-06 |
| 28255 | N64.1 | UK | Europe | throat swab | carrier | Neisseria meningitidis | Y | ERR144497 | 2014-03-06 |
| 28256 | N117.1 | UK | Europe | throat swab | carrier | Neisseria meningitidis | Y | ERR144498 | 2014-03-06 |
| 28257 | N124.1 | UK | Europe | throat swab | carrier | Neisseria meningitidis | Y | ERR144499 | 2014-03-06 |
| 28258 | N128.1 | UK | Europe | throat swab | carrier | Neisseria meningitidis |  | ERR144500 | 2014-03-06 |
| 28259 | N258.1 | UK | Europe | throat swab | carrier | Neisseria meningitidis | Y | ERR144501 | 2014-03-06 |
| 28260 | N264.1 | UK | Europe | throat swab | carrier | Neisseria meningitidis | Y | ERR144502 | 2014-03-06 |
| 28261 | N259.1 | UK | Europe | throat swab | carrier | Neisseria meningitidis | Y | ERR144503 | 2014-03-06 |
| 28262 | N222.1 | UK | Europe | throat swab | carrier | Neisseria meningitidis | Y | ERR144504 | 2014-03-06 |
| 28263 | N114.1 | UK | Europe | throat swab | carrier | Neisseria meningitidis | E | ERR144505 | 2014-03-06 |
| 28264 | N134.1 | UK | Europe | throat swab | carrier | Neisseria meningitidis |  | ERR144506 | 2014-03-06 |
| 28265 | N185.1 | UK | Europe | throat swab | carrier | Neisseria meningitidis |  | ERR144507 | 2014-03-06 |
| 28266 | N185.2 | UK | Europe | throat swab | carrier | Neisseria meningitidis |  | ERR144508 | 2014-03-06 |
| 28267 | N262.1 | UK | Europe | throat swab | carrier | Neisseria meningitidis |  | ERR144509 | 2014-03-06 |
| 28268 | N73.1 | UK | Europe | throat swab | carrier | Neisseria meningitidis |  | ERR144510 | 2014-03-06 |
| 28269 | N188.1 | UK | Europe | throat swab | carrier | Neisseria meningitidis | Y | ERR144511 | 2014-03-06 |
| 28270 | N199.1 | UK | Europe | throat swab | carrier | Neisseria meningitidis |  | ERR144512 | 2014-03-06 |
| 28271 | N176.1 | UK | Europe | throat swab | carrier | Neisseria meningitidis |  | ERR144513 | 2014-03-06 |
| 28272 | N86.1 | UK | Europe | throat swab | carrier | Neisseria meningitidis |  | ERR144514 | 2014-03-06 |
| 28273 | N349.1 | UK | Europe | throat swab | carrier | Neisseria meningitidis | Y | ERR144515 | 2014-03-06 |
| 28274 | N424.1 | UK | Europe | throat swab | carrier | Neisseria meningitidis | Y | ERR144516 | 2014-03-06 |
| 28275 | N342.1 | UK | Europe | throat swab | carrier | Neisseria meningitidis | Y | ERR144517 | 2014-03-06 |
| 28276 | N343.1 | UK | Europe | throat swab | carrier | Neisseria meningitidis | Y | ERR144518 | 2014-03-06 |
| 28277 | N429.1 | UK | Europe | throat swab | carrier | Neisseria meningitidis | Y | ERR144519 | 2014-03-06 |
| 28278 | N438.1 | UK | Europe | throat swab | carrier | Neisseria meningitidis | Y | ERR144520 | 2014-03-06 |
| 28279 | N449.1 | UK | Europe | throat swab | carrier | Neisseria meningitidis | Y | ERR144521 | 2014-03-06 |
| 28280 | N331.1 | UK | Europe | throat swab | carrier | Neisseria meningitidis | Y | ERR144522 | 2014-03-06 |
| 28281 | N348.1 | UK | Europe | throat swab | carrier | Neisseria meningitidis | Y | ERR144523 | 2014-03-06 |
| 28282 | N417.1 | UK | Europe | throat swab | carrier | Neisseria meningitidis | NG | ERR144524 | 2014-03-06 |
| 28283 | N336.1 | UK | Europe | throat swab | carrier | Neisseria meningitidis | Y | ERR144525 | 2014-03-06 |
| 28284 | N420.1 | UK | Europe | throat swab | carrier | Neisseria meningitidis |  | ERR144526 | 2014-03-06 |
| 28285 | N431.1 | UK | Europe | throat swab | carrier | Neisseria meningitidis | Y | ERR144527 | 2014-03-06 |
| 28286 | N359.1 | UK | Europe | throat swab | carrier | Neisseria meningitidis |  | ERR144528 | 2014-03-06 |
| 28287 | N445.1 | UK | Europe | throat swab | carrier | Neisseria meningitidis | Y | ERR144529 | 2014-03-06 |
| 28288 | N459.1 | UK | Europe | throat swab | carrier | Neisseria meningitidis | Y | ERR144530 | 2014-03-06 |
| 28289 | N330.1 | UK | Europe | throat swab | carrier | Neisseria meningitidis |  | ERR144531 | 2014-03-06 |
| 28290 | N333.1 | UK | Europe | throat swab | carrier | Neisseria meningitidis |  | ERR144532 | 2014-03-06 |
| 28291 | N456.1 | UK | Europe | throat swab | carrier | Neisseria meningitidis |  | ERR144533 | 2014-03-06 |
| 28292 | N456.2 | UK | Europe | throat swab | carrier | Neisseria meningitidis |  | ERR144534 | 2014-03-06 |
| 28293 | N446.1 | UK | Europe | throat swab | carrier | Neisseria meningitidis |  | ERR144535 | 2014-03-06 |
| 28294 | N450.1 | UK | Europe | throat swab | carrier | Neisseria meningitidis |  | ERR144536 | 2014-03-06 |
| 28295 | N462.1 | UK | Europe | throat swab | carrier | Neisseria meningitidis | Y | ERR144537 | 2014-03-06 |
| 28296 | N378.1 | UK | Europe | throat swab | carrier | Neisseria meningitidis |  | ERR144538 | 2014-03-06 |
| 28297 | N408.1 | UK | Europe | throat swab | carrier | Neisseria meningitidis |  | ERR144539 | 2014-03-06 |
| 28298 | N447.1 | UK | Europe | throat swab | carrier | Neisseria meningitidis |  | ERR144540 | 2014-03-06 |
| 28299 | N59.1.1 | UK | Europe |  |  | Neisseria meningitidis |  | ERR346691 | 2014-03-10 |
| 28300 | N59.3 | UK | Europe |  |  | Neisseria meningitidis |  | ERR346692 | 2014-03-10 |
| 28301 | N59.4 | UK | Europe |  |  | Neisseria meningitidis |  | ERR346693 | 2014-03-10 |
| 28302 | N59.5 | UK | Europe |  |  | Neisseria meningitidis |  | ERR346694 | 2014-03-10 |
| 28303 | N59.6 | UK | Europe |  |  | Neisseria meningitidis |  | ERR346695 | 2014-03-10 |
| 28304 | N59.7 | UK | Europe |  |  | Neisseria meningitidis |  | ERR346696 | 2014-03-10 |
| 28305 | N59.8 | UK | Europe |  |  | Neisseria meningitidis |  | ERR346697 | 2014-03-10 |
| 28306 | N59.9 | UK | Europe |  |  | Neisseria meningitidis |  | ERR349236 | 2014-03-10 |
| 28307 | N59.10 | UK | Europe |  |  | Neisseria meningitidis |  | ERR349237 | 2014-03-10 |
| 28308 | N59.11 | UK | Europe |  |  | Neisseria meningitidis |  | ERR346698 | 2014-03-10 |
| 28309 | N253.1 | UK | Europe |  |  | Neisseria meningitidis |  | ERR346699 | 2014-03-10 |
| 28310 | N253.2 | UK | Europe |  |  | Neisseria meningitidis |  | ERR346700 | 2014-03-10 |
| 28311 | N253.3 | UK | Europe |  |  | Neisseria meningitidis |  | ERR346701 | 2014-03-10 |
| 28312 | N253.4 | UK | Europe |  |  | Neisseria meningitidis |  | ERR346702 | 2014-03-10 |
| 28313 | N253.5 | UK | Europe |  |  | Neisseria meningitidis |  | ERR346703 | 2014-03-10 |
| 28314 | N253.6 | UK | Europe |  |  | Neisseria meningitidis |  | ERR346704 | 2014-03-10 |
| 28315 | N253.7 | UK | Europe |  |  | Neisseria meningitidis |  | ERR346705 | 2014-03-10 |
| 28316 | N253.8 | UK | Europe |  |  | Neisseria meningitidis |  | ERR346706 | 2014-03-10 |
| 28317 | N253.9 | UK | Europe |  |  | Neisseria meningitidis |  | ERR346707 | 2014-03-10 |
| 28318 | N253.10 | UK | Europe |  |  | Neisseria meningitidis |  | ERR346708 | 2014-03-10 |
| 28319 | N352.1 | UK | Europe |  |  | Neisseria meningitidis |  | ERR346709 | 2014-03-10 |
| 28320 | N352.2 | UK | Europe |  |  | Neisseria meningitidis |  | ERR346710 | 2014-03-10 |
| 28321 | N352.3 | UK | Europe |  |  | Neisseria meningitidis |  | ERR346711 | 2014-03-10 |
| 28322 | N352.4 | UK | Europe |  |  | Neisseria meningitidis |  | ERR346712 | 2014-03-10 |
| 28323 | N352.5 | UK | Europe |  |  | Neisseria meningitidis |  | ERR346713 | 2014-03-10 |
| 28324 | N352.6 | UK | Europe |  |  | Neisseria meningitidis |  | ERR346714 | 2014-03-10 |
| 28325 | N352.7 | UK | Europe |  |  | Neisseria meningitidis |  | ERR346715 | 2014-03-10 |
| 28326 | N352.8 | UK | Europe |  |  | Neisseria meningitidis |  | ERR346716 | 2014-03-10 |
| 28327 | N352.9 | UK | Europe |  |  | Neisseria meningitidis |  | ERR346717 | 2014-03-10 |
| 28328 | N352.10 | UK | Europe |  |  | Neisseria meningitidis |  | ERR346718 | 2014-03-10 |
| 28329 | N438.1.1 | UK | Europe |  |  | Neisseria meningitidis |  | ERR346719 | 2014-03-10 |
| 28330 | N438.2 | UK | Europe |  |  | Neisseria meningitidis |  | ERR346720 | 2014-03-10 |
| 28331 | N438.3 | UK | Europe |  |  | Neisseria meningitidis |  | ERR346721 | 2014-03-10 |
| 28332 | N438.4 | UK | Europe |  |  | Neisseria meningitidis |  | ERR346722 | 2014-03-10 |
| 28333 | N438.5 | UK | Europe |  |  | Neisseria meningitidis |  | ERR346723 | 2014-03-10 |
| 28334 | N438.6 | UK | Europe |  |  | Neisseria meningitidis |  | ERR346724 | 2014-03-10 |
| 28335 | N438.7 | UK | Europe |  |  | Neisseria meningitidis |  | ERR346725 | 2014-03-10 |
| 28336 | N438.8 | UK | Europe |  |  | Neisseria meningitidis |  | ERR346726 | 2014-03-10 |
| 28337 | N438.9 | UK | Europe |  |  | Neisseria meningitidis |  | ERR346727 | 2014-03-10 |
| 28338 | N438.10 | UK | Europe |  |  | Neisseria meningitidis |  | ERR346728 | 2014-03-10 |

**Supplementary table 2. Genomes deposited by Bayliss as of 22.02.17.** Genomes are available within the pubMLST *Neisseria ssp.* database.
