# Supplementary material for: Phasome analysis of pathogenic and commensal Neisseria species expands the known repertoire of phase variable genes, and highlights common adaptive strategies
Source: PLoS One. 2018 May 15;13(5):e0196675. doi: 10.1371/journal.pone.0196675 (PMC5953494; doi:10.1371/journal.pone.0196675)
Supplement: S3 Table — Statistical significance were determined by an ordinary one way ANOVA, with Tukey’s multiple comparisons. (DOCX) [file pone.0196675.s003.docx]

| Tukey's multiple comparisons test | Mean Diff. | 95.00% CI of diff. | Significant? | Summary | Adjusted P Value |
| --- | --- | --- | --- | --- | --- |
|  |  |  |  |  |  |
| *N. meningitidis* vs. *N. gonnorheae* | -14.76 | -25.44 to -4.076 | Yes | *** | 0.0005 |
| *N. meningitidis* vs. *N. bacilliformis* | 15.24 | -6.158 to 36.65 | No | ns | 0.4642 |
| *N. meningitidis* vs. *N. cinerea* | 9.844 | -9.415 to 29.1 | No | ns | 0.8964 |
| *N. meningitidis* vs. *N. elongata* | 18.24 | -3.158 to 39.65 | No | ns | 0.1865 |
| *N. meningitidis* vs. *N. flavesens* | 23.67 | 7.201 to 40.14 | Yes | *** | 0.0002 |
| *N. meningitidis* vs. *N. lactamica* | -9.856 | -23.88 to 4.166 | No | ns | 0.4868 |
| *N. meningitidis* vs. *N. mucosa* | 21.64 | 2.385 to 40.9 | Yes | * | 0.0131 |
| *N. meningitidis* vs. *N. polysaccharea* | 13.74 | -1.754 to 29.24 | No | ns | 0.1415 |
| *N. meningitidis* vs. *N. shaygennii* | 25.24 | -16.77 to 67.26 | No | ns | 0.7335 |
| *N. meningitidis* vs. *N. sicca* | 29.96 | 13.49 to 46.43 | Yes | **** | <0.0001 |
| *N. meningitidis* vs. *N. subflava* | 19.24 | -0.01492 to 38.5 | No | ns | 0.0504 |
| *N. meningitidis* vs. *N. wadsworthii* | 32.24 | -9.769 to 74.26 | No | ns | 0.3384 |
| *N. meningitidis* vs. *N. weaveri* | 32.64 | 13.39 to 51.9 | Yes | **** | <0.0001 |
| *N. gonnorheae* vs. *N. bacilliformis* | 30 | 7.035 to 52.97 | Yes | ** | 0.0013 |
| *N. gonnorheae* vs. *N. cinerea* | 24.6 | 3.617 to 45.58 | Yes | ** | 0.0074 |
| *N. gonnorheae* vs. *N. elongata* | 33 | 10.03 to 55.97 | Yes | *** | 0.0002 |
| *N. gonnorheae* vs. *N. flavesens* | 38.43 | 19.97 to 56.89 | Yes | **** | <0.0001 |
| *N. gonnorheae* vs. *N. lactamica* | 4.9 | -11.41 to 21.21 | No | ns | 0.9990 |
| *N. gonnorheae* vs. *N. mucosa* | 36.4 | 15.42 to 57.38 | Yes | **** | <0.0001 |
| *N. gonnorheae* vs. *N. polysaccharea* | 28.5 | 10.91 to 46.09 | Yes | **** | <0.0001 |
| *N. gonnorheae* vs. *N. shaygennii* | 40 | -2.831 to 82.83 | No | ns | 0.0939 |
| *N. gonnorheae* vs. *N. sicca* | 44.71 | 26.26 to 63.17 | Yes | **** | <0.0001 |
| *N. gonnorheae* vs. *N. subflava* | 34 | 13.02 to 54.98 | Yes | **** | <0.0001 |
| *N. gonnorheae* vs. *N. wadsworthii* | 47 | 4.169 to 89.83 | Yes | * | 0.0177 |
| *N. gonnorheae* vs. *N. weaveri* | 47.4 | 26.42 to 68.38 | Yes | **** | <0.0001 |
| *N. bacilliformis* vs. *N. cinerea* | -5.4 | -33.4 to 22.6 | No | ns | >0.9999 |
| *N. bacilliformis* vs. *N. elongata* | 3 | -26.52 to 32.52 | No | ns | >0.9999 |
| *N. bacilliformis* vs. *N. flavesens* | 8.429 | -17.74 to 34.59 | No | ns | 0.9980 |
| *N. bacilliformis* vs. *N. lactamica* | -25.1 | -49.8 to -0.4027 | Yes | * | 0.0424 |
| *N. bacilliformis* vs. *N. mucosa* | 6.4 | -21.6 to 34.4 | No | ns | >0.9999 |
| *N. bacilliformis* vs. *N. polysaccharea* | -1.5 | -27.06 to 24.06 | No | ns | >0.9999 |
| *N. bacilliformis* vs. *N. shaygennii* | 10 | -36.67 to 56.67 | No | ns | >0.9999 |
| *N. bacilliformis* vs. *N. sicca* | 14.71 | -11.45 to 40.88 | No | ns | 0.8131 |
| *N. bacilliformis* vs. *N. subflava* | 4 | -24 to 32 | No | ns | >0.9999 |
| *N. bacilliformis* vs. *N. wadsworthii* | 17 | -29.67 to 63.67 | No | ns | 0.9933 |
| *N. bacilliformis* vs. *N. weaveri* | 17.4 | -10.6 to 45.4 | No | ns | 0.6866 |
| *N. cinerea* vs. *N. elongata* | 8.4 | -19.6 to 36.4 | No | ns | 0.9990 |
| *N. cinerea* vs. *N. flavesens* | 13.83 | -10.62 to 38.27 | No | ns | 0.8066 |
| *N. cinerea* vs. *N. lactamica* | -19.7 | -42.57 to 3.165 | No | ns | 0.1737 |
| *N. cinerea* vs. *N. mucosa* | 11.8 | -14.6 to 38.2 | No | ns | 0.9609 |
| *N. cinerea* vs. *N. polysaccharea* | 3.9 | -19.9 to 27.7 | No | ns | >0.9999 |
| *N. cinerea* vs. *N. shaygennii* | 15.4 | -30.33 to 61.13 | No | ns | 0.9968 |
| *N. cinerea* vs. *N. sicca* | 20.11 | -4.33 to 44.56 | No | ns | 0.2328 |
| *N. cinerea* vs. *N. subflava* | 9.4 | -17 to 35.8 | No | ns | 0.9946 |
| *N. cinerea* vs. *N. wadsworthii* | 22.4 | -23.33 to 68.13 | No | ns | 0.9226 |
| *N. cinerea* vs. *N. weaveri* | 22.8 | -3.603 to 49.2 | No | ns | 0.1709 |
| *N. elongata* vs. *N. flavesens* | 5.429 | -20.74 to 31.59 | No | ns | >0.9999 |
| *N. elongata* vs. *N. lactamica* | -28.1 | -52.8 to -3.403 | Yes | * | 0.0111 |
| *N. elongata* vs. *N. mucosa* | 3.4 | -24.6 to 31.4 | No | ns | >0.9999 |
| *N. elongata* vs. *N. polysaccharea* | -4.5 | -30.06 to 21.06 | No | ns | >0.9999 |
| *N. elongata* vs. *N. shaygennii* | 7 | -39.67 to 53.67 | No | ns | >0.9999 |
| *N. elongata* vs. *N. sicca* | 11.71 | -14.45 to 37.88 | No | ns | 0.9604 |
| *N. elongata* vs. *N. subflava* | 1 | -27 to 29 | No | ns | >0.9999 |
| *N. elongata* vs. *N. wadsworthii* | 14 | -32.67 to 60.67 | No | ns | 0.9990 |
| *N. elongata* vs. *N. weaveri* | 14.4 | -13.6 to 42.4 | No | ns | 0.8922 |
| *N. flavesens* vs. *N. lactamica* | -33.53 | -54.1 to -12.96 | Yes | **** | <0.0001 |
| *N. flavesens* vs. *N. mucosa* | -2.029 | -26.47 to 22.42 | No | ns | >0.9999 |
| *N. flavesens* vs. *N. polysaccharea* | -9.929 | -31.53 to 11.68 | No | ns | 0.9515 |
| *N. flavesens* vs. *N. shaygennii* | 1.571 | -43.06 to 46.2 | No | ns | >0.9999 |
| *N. flavesens* vs. *N. sicca* | 6.286 | -16.03 to 28.6 | No | ns | 0.9995 |
| *N. flavesens* vs. *N. subflava* | -4.429 | -28.87 to 20.02 | No | ns | >0.9999 |
| *N. flavesens* vs. *N. wadsworthii* | 8.571 | -36.06 to 53.2 | No | ns | >0.9999 |
| *N. flavesens* vs. *N. weaveri* | 8.971 | -15.47 to 33.42 | No | ns | 0.9928 |
| *N. lactamica* vs. *N. mucosa* | 31.5 | 8.635 to 54.37 | Yes | *** | 0.0005 |
| *N. lactamica* vs. *N. polysaccharea* | 23.6 | 3.798 to 43.4 | Yes | ** | 0.0058 |
| *N. lactamica* vs. *N. shaygennii* | 35.1 | -8.684 to 78.88 | No | ns | 0.2704 |
| *N. lactamica* vs. *N. sicca* | 39.81 | 19.24 to 60.39 | Yes | **** | <0.0001 |
| *N. lactamica* vs. *N. subflava* | 29.1 | 6.235 to 51.97 | Yes | ** | 0.0021 |
| *N. lactamica* vs. *N. wadsworthii* | 42.1 | -1.684 to 85.88 | No | ns | 0.0727 |
| *N. lactamica* vs. *N. weaveri* | 42.5 | 19.63 to 65.37 | Yes | **** | <0.0001 |
| *N. mucosa* vs. *N. polysaccharea* | -7.9 | -31.7 to 15.9 | No | ns | 0.9973 |
| *N. mucosa* vs. *N. shaygennii* | 3.6 | -42.13 to 49.33 | No | ns | >0.9999 |
| *N. mucosa* vs. *N. sicca* | 8.314 | -16.13 to 32.76 | No | ns | 0.9965 |
| *N. mucosa* vs. *N. subflava* | -2.4 | -28.8 to 24 | No | ns | >0.9999 |
| *N. mucosa* vs. *N. wadsworthii* | 10.6 | -35.13 to 56.33 | No | ns | >0.9999 |
| *N. mucosa* vs. *N. weaveri* | 11 | -15.4 to 37.4 | No | ns | 0.9779 |
| *N. polysaccharea* vs. *N. shaygennii* | 11.5 | -32.78 to 55.78 | No | ns | 0.9998 |
| *N. polysaccharea* vs. *N. sicca* | 16.21 | -5.391 to 37.82 | No | ns | 0.3753 |
| *N. polysaccharea* vs. *N. subflava* | 5.5 | -18.3 to 29.3 | No | ns | >0.9999 |
| *N. polysaccharea* vs. *N. wadsworthii* | 18.5 | -25.78 to 62.78 | No | ns | 0.9774 |
| *N. polysaccharea* vs. *N. weaveri* | 18.9 | -4.899 to 42.7 | No | ns | 0.2846 |
| *N. shaygennii* vs. *N. sicca* | 4.714 | -39.91 to 49.34 | No | ns | >0.9999 |
| *N. shaygennii* vs. *N. subflava* | -6 | -51.73 to 39.73 | No | ns | >0.9999 |
| *N. shaygennii* vs. *N. wadsworthii* | 7 | -52.04 to 66.04 | No | ns | >0.9999 |
| *N. shaygennii* vs. *N. weaveri* | 7.4 | -38.33 to 53.13 | No | ns | >0.9999 |
| *N. sicca* vs. *N. subflava* | -10.71 | -35.16 to 13.73 | No | ns | 0.9665 |
| *N. sicca* vs. *N. wadsworthii* | 2.286 | -42.34 to 46.91 | No | ns | >0.9999 |
| *N. sicca* vs. *N. weaveri* | 2.686 | -21.76 to 27.13 | No | ns | >0.9999 |
| *N. subflava* vs. *N. wadsworthii* | 13 | -32.73 to 58.73 | No | ns | 0.9994 |
| *N. subflava* vs. *N. weaveri* | 13.4 | -13 to 39.8 | No | ns | 0.9012 |
| *N. wadsworthii* vs. *N. weaveri* | 0.4 | -45.33 to 46.13 | No | ns | >0.9999 |

**Supplementary table 3. Statistical analysis of the number of PV genes detected in each species.** An ordinary one way ANOVE with Tukey’s multiple comparisons was performed.
